# Nociceptor-expressed ephrin-B2 regulates inflammatory and neuropathic pain

**DOI:** 10.1186/1744-8069-6-77

**Published:** 2010-11-08

**Authors:** Jing Zhao, Guanglu Yuan, Cruz M Cendan, Mohammed A Nassar, Malin C Lagerström, Klas Kullander, Isabella Gavazzi, John N Wood

**Affiliations:** 1 Molecular Nociception Group, Wolfson Institute for Biomedical Research (WIBR), Cruciform Building, University College London (UCL), London WC1E 6BT, UK; 2 Department of Pharmacology and Institute of Neuroscience, Faculty of Medicine, University of Granada, Avenida de Madrid 11, 18012 Granada, Spain; 3 Unit of Developmental Genetics, Department of Neuroscience, Uppsala University, Biomedical Center, Box 593, Husargatan 1, 75123 Uppsala, Sweden; 4 Wolfson Centre for Age Related Diseases (CARD), Hodgkin Building, Wolfson Wing, King's College London (KCL), London SE1 1UL, UK

## Abstract

**Background:**

EphB receptors and their ephrin-B ligands play an important role in nervous system development, as well as synapse formation and plasticity in the adult brain. Recent studies show that intrathecal treatment with EphB-receptor activator ephrinB2-Fc induced thermal hyperalgesia and mechanical allodynia in rat, indicating that ephrin-B2 in small dorsal root ganglia (DRG) neurons and EphB receptors in the spinal cord modulate pain processing. To examine the role of ephrin-B2 in peripheral pain pathways, we deleted ephrin-B2 in Nav1.8+ nociceptive sensory neurons with the Cre-loxP system. Sensory neuron numbers and terminals were examined using neuronal makers. Pain behavior in acute, inflammatory and neuropathic pain models was assessed in the ephrin-B2 conditional knockout (CKO) mice. We also investigated the c-Fos expression and NMDA receptor NR2B phosphorylation in ephrin-B2 CKO mice and littermate controls.

**Results:**

The ephrin-B2 CKO mice were healthy with no sensory neuron loss. However, pain-related behavior was substantially altered. Although acute pain behavior and motor co-ordination were normal, inflammatory pain was attenuated in ephrin-B2 mutant mice. Complete Freund's adjuvant (CFA)-induced mechanical hyperalgesia was halved. Formalin-induced pain behavior was attenuated in the second phase, and this correlated with diminished tyrosine phosphorylation of N-methyl-D-aspartic acid (NMDA) receptor subunit NR2B in the dorsal horn. Thermal hyperalgesia and mechanical allodynia were significantly reduced in the Seltzer model of neuropathic pain.

**Conclusions:**

Presynaptic ephrin-B2 expression thus plays an important role in regulating inflammatory pain through the regulation of synaptic plasticity in the dorsal horn and is also involved in the pathogenesis of some types of neuropathic pain.

## Background

The Eph receptors and their ephrin ligands, the ephrins, are the largest family of receptor tyrosine kinases. The interactions between Eph receptors and their ligands, classified into A and B-subclasses based on sequence homology and binding affinity, can initiate bidirectional signaling [1,2]. Eph receptors have diverse activities on both neuronal and non-neuronal cells and influence cell-substrate adhesion, intercellular junctions, cell shape and cell movement [3]. Eph receptors play essential roles in nervous system circuit assembly during development [4,5] and regulate synaptic function mediated by NMDA receptors in the adult brain [6]. Several studies demonstrated that EphB receptors and ephrins play key roles as modulators of synaptic plasticity in the central nervous system [7,8].

Recent work using neutralizing receptor bodies (EphB1/Fc fragments) or stabilized activators (ephrin-B2/Fc) suggests that Eph receptors and their ligands also play an important role in pain signaling between DRG and neurons of the dorsal horn of spinal cord [9]. Ephs/ephrins are also involved in neuropathic pain processing. Intrathecal administration of ephrin-B2 siRNA decreased the expression of ephrin-B2 and mechanical allodynia after sciatic nerve crush [10]. Song et al. showed that expression of both ephrin-B1 and EphB1 are increased in the DRG and spinal cord after chronic constriction injury and dorsal rhizotomy or a combination of both [11]. EphB1/Fc and EphB2/Fc administration also prevented hyperexcitability of nociceptive neurons in the DRG and sensitization of wide dynamic range neurons in the dorsal horn in a neuropathic pain model in rat [12]. They later identified EphB1 as the specific EphB receptor involved in both neuropathic pain and morphine tolerance dependence using EphB1 knockout mice [13]. They also demonstrated that EphB1 is essential for long-term potentiation between primary afferent c-fibres and dorsal horn neurons in the spinal cord [14]. Although these studies suggest that EphB receptors and their ligands (ephrin-B1 and/or ephrin-B2) are involved in pain processing in the DRG and spinal cord, the cell types involved and mechanisms are still not clear. Ephrin-B1 global null mice are lethal [15]. The signaling mechanisms based on the administration of ectopic EphB/Fc and ephrin-B2/Fc chimerae remain uncertain, because over-expression studies may be unphysiological, whilst blocking receptor bodies may not completely inhibit signaling.

In the present study, we have investigated the role of ephrin-B2 mediated signaling in pain pathways by deleting ephrin-B2 from Nav1.8-expressing nociceptors with the Cre-recombinase-loxP system. By crossing two floxed ephrin-B2 strains, a floxed exon 1 mouse [16] and a floxed exon 2 mouse [17] with the Nav1.8 promoter-driven Cre mouse Nav1.8-Cre [18], we generated ephrin-B2 CKO mice, as the global ephrin-B2 homozygous mutant mice die at E9.5 with severe cardiovascular defects [19,20]. Here we present an analysis of signaling to the central nervous system and pain behavior in the nociceptor-specific ephrin-B2 null mice.

## Results

### Floxed exon 2 ephrin-B2 is deleted in nociceptors by Nav1.8-Cre

To generate ephrin-B2 CKO mice, we crossed a floxed exon 1 ephrin-B2 mouse [16] with the Nav1.8-Cre mouse line. We genotyped the CKO and littermate controls by PCR. The result shows that the knockout PCR product was very weak from conditional null mutant DRG. However, heterozygous global null ephrin-B2 mice derived from crossing with a ROSA-Cre deletor showed a very strong appropriate PCR product (Additional file 1, S1). The Cre recombinase gene in the ephrin-B2 CKO mice was confirmed with PCR and Southern blot (Additional file 1, S2). We therefore examined the floxed exon 1 knockout locus with Southern blots. The result shows that the exon 1 ephrin-B2 knockout bands (6.1 kb/*Hind*III and 4.8 kb/*Hind*III/*Eco*RI) were clearly revealed from ephrin-B2 global knockout mice, but were not found in the exon 1 ephrin-B2 CKO mice (Additional file 1, S3). In terms of PCR and Southern blot results, the deletion of exon 1 occured in DRG, but the efficiency was very low, probably because the Nav.1.8 promoter drives Cre expression much less efficiently than the global Cre promoter, and some regions adjacent to exon 1 may be masked by regulatory chromatin proteins.

**Figure 1:**
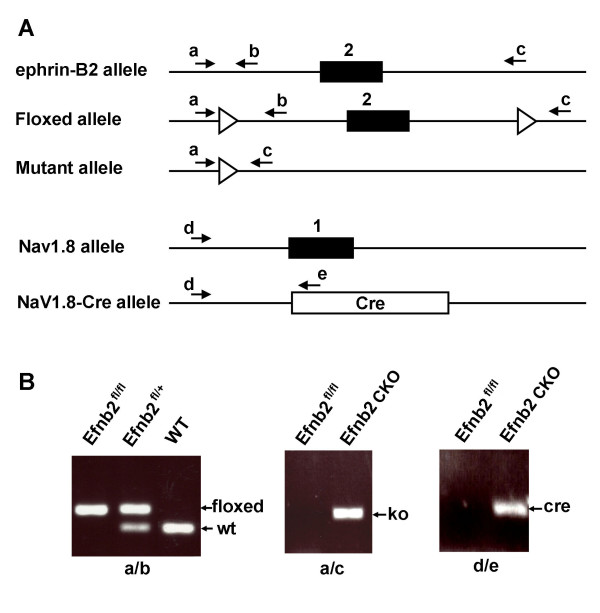
**Exon 2 of ephrin-B2 was deleted in DRG in Efnb2 CKO mice**. **(A) **Diagram showing the ephrin-B2 wild-type locus, the targeted locus before and after Cre excision, Nav1.8 wild-type and Nav1.8-Cre locus. Exons are represented as numbered boxes. The loxP site (blank triangles) and the PCR primers (black arrows) are indicated. **(B) **Genotyping analysis with PCR. The DRGs genomic DNA from Efnb2 CKO mice, Efnb2^fl/fl ^controls, heterozygous floxed exon 2 ephrin-B2 (Efnb2^fl/+^) mice and C57BL/6 wild-type (WT) mice were examined with PCR. The wild-type band (wt), floxed band (floxed) and Cre band (cre) were amplified with primer sets a/b, a/c and d/e respectively. The knockout band (ko) was found only from Efnb2 CKO mutant mice.

We therefore tested a floxed exon 2 ephrin-B2 mouse [17], and crossed the mouse with Nav1.8-Cre. Figure 1A shows the ephrin-B2 locus, loxP sites and position of PCR primers. Using PCR analysis, we examined the Cre excision in CKO mice. The result shows that knockout band (400 bp) was clearly apparent from DRG DNA in the CKO mice (Figure 1B). We therefore used the exon 2 ephrin-B2 conditional knockout (Efnb2 CKO) mutant mice and their floxed exon 2 ephrin-B2 (Efnb2^fl/fl^) littermate controls for further experiments.

### Ephrin-B2 deletion does not affect either the survival of DRG neurons or distribution of the nociceptive primary afferent terminals in the spinal cord

**Figure 2:**
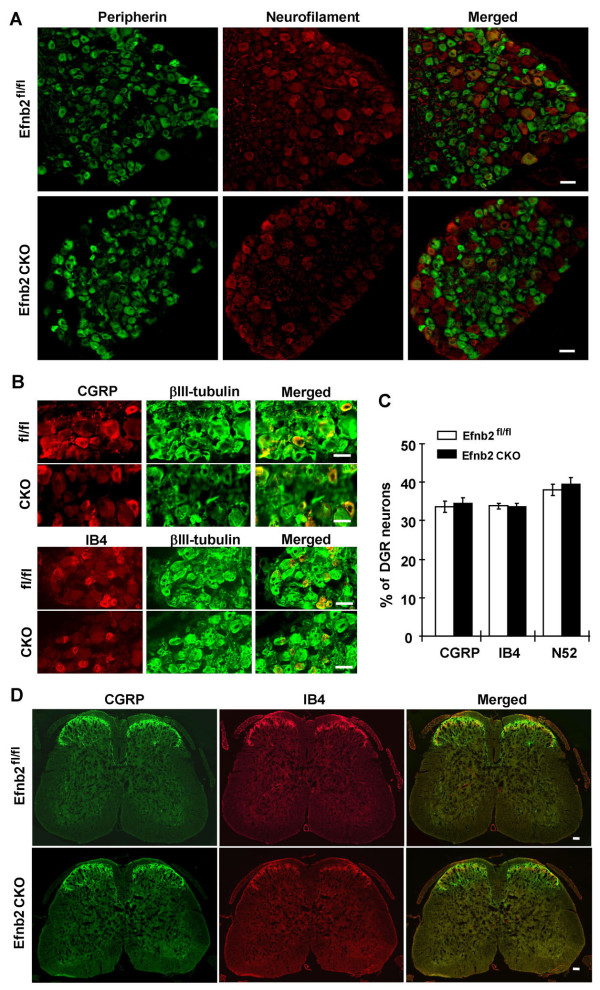
**Neural marker expression was normal in DRG neurons of ephrin-B2 CKO mice**. **(A) **Whole DRG sections of Efnb2 CKO mutant mice and Efnb2^fl/fl ^littermate controls were labeled with anti-peripherin (green) and anti-neurofilament (red) antibodies. The right panels are merged images of the left and middle panels. **(B) **DRG sections were also labeled with anti-CGRP (red) and anti-IB4 (red) antibodies, co-labeled with anti-βIII-tubulin (green). The right panels are erged images of the left and middle panels. **(C) **The proportions of CGRP, IB4 and neurofilament (N52) expressing neurons were normal in Efnb2 CKO animals compared to Efnb2^fl/fl ^littermate controls. **(D) **Cross section of lumbar spinal cord (L3 - L5) stained with anti-CGRP (green) antibody and anti-IB4 (red) antibody. In laminae I-II, both CGRP positive terminals and IB4 positive terminals were identified in the Efnb2 CKO mutant mice and Efnb2^fl/fl ^littermate controls. Scale bar = 50 μm.

Whole DRG sections were examined to test for effects of ephrin-B2 deletion on neuronal survival. The sections were labeled for neuronal markers peripherin and neurofilament heavy chain. The result shows that most small and medium diameter DRG neurons (nociceptors) were labeled with anti-peripherin antibody in green, large diameter DRG neurons were labeled with anti-neurofilament (N200) in red (Figure 2A). There was no apparent difference between Efnb2^fl/fl ^controls and Efnb2 CKO mutant mice. The DRG sections from L5 were also labeled with neuronal marker IB4, anti-CGRP and anti-neurofilament (N52), and co-labeled with neuron marker βIII tubulin. All the sections from Efnb2 CKO mutants had a normal appearance, with no significant difference from Efnb2^fl/fl ^controls (Figure 2B), and the proportions of IB4, CGRP and neurofilament (N52) positive neurons in Efnb2 CKO mice ware similar to the Efnb2^fl/fl ^controls (Figure 2C). The spinal cord sections from lumber 4-5, labeled with IB4 and anti-CGRP, were used to investigate the distribution of the nociceptive primary afferent terminals from Efnb2 CKO mutant mice. The result shows that no significant difference was found on estimated area of termination or their staining intensity from the IB4 and CGRP labeling (Figure 2D).

### Ephrin-B2 CKO mutant mice show no gross abnormalities

**Figure 3:**
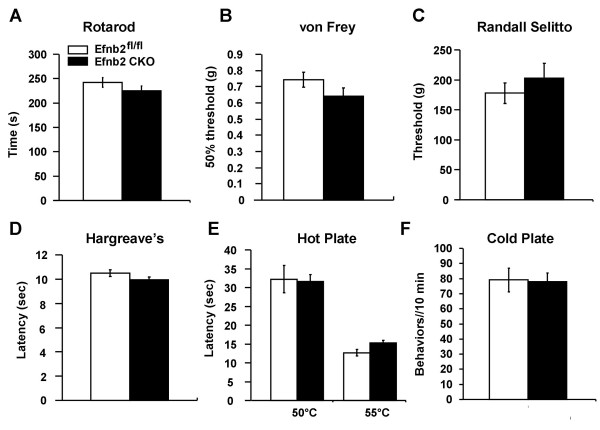
**Acute pain behavior was not affected in ephrin-B2 mutant mice**. **(A) **Rotarod studies showed no motor deficits in Efnb2 CKO animals. **(B) **Responses to low-threshold mechanical stimulation by von Frey filaments are normal in Efnb2 CKO mutant mice. **(C) **Acute mechanical pressure applied with the Randall Selitto apparatus demonstrated identical behavior in Efnb2 CKO and Efnb2^fl/fl ^mice. **(D) **Hargreaves' apparatus also demonstrates identical latencies of response to thermal stimulation in Efnb2 CKO and Efnb2^fl/fl ^mice. **(E) **Supra-spinal reflexes to heat (50 and 55°C) using the hot plate apparatus demonstrates identical latencies in Efnb2 CKO and Efnb2^fl/fl ^mice. **(F) **Cold behavior (number of liftings) is the same in Efnb2^fl/fl ^and Efnb2 CKO mice (0°C) using the cold plate apparatus.

Efnb2 CKO mutants were healthy, fertile, and apparently normal. They showed no obvious differences from Efnb2^fl/fl ^controls in appearance and spontaneous behavior. Age (8-13 weeks) -matched and sex-mixed Efnb2 CKO mutant mice (n = 9) and Efnb2^fl/fl ^controls (n = 8) were weighed and used for von Frey, Randall Selitto, Hargreaves', hot plate and cold plate tests. The average weight was 26.87 ± 1.88 gram (g) (Efnb2 CKO) and 28.4 ± 1.47 g (Efnb2^fl/fl^). Motor function of the mice was examined with a rotarod. The average time that animals stay on the rod was 241.9 ± 9.8 seconds (sec) (Efnb2^fl/fl^) and 224.6 ± 10.4 sec (Efnb2 CKO). There were no significant differences between the two groups either in the weight (t-test, p = 0.27) or in the time spent on the rod (t-test, p = 0.13) (Figure 3A).

### Ephrin-B2 CKO mutant mice show normal acute pain behavior

The response to mechanical stimulation was tested by using von Frey hairs. 50% threshold of paw withdrawal in response to von Frey hairs was 0.74 ± 0.04 g (Efnb2^fl/fl^) and 0.64 ± 0.05 g (Efnb2 CKO). There was no significant difference between these two groups (t-test, p = 0.16) (Figure 3B). To assess the response to noxious mechanical pressure, the animals were tested with Randall-Selitto apparatus. The mean of weight that resulted in a withdrawal response was 179.3 ± 17.2 g (Efnb2^fl/fl^) and 202.7 ± 25.2 g (Efnb2 CKO). No significant difference in weight applied to the tail was found (t-test, p = 0.43) (Figure 3C). Acute thermal threshold was examined using Hargreaves' test. The mean latency of withdrawal of the hindpaw from a thermal stimulus was 10.5 ± 0.2 sec (Efnb2^fl/fl^) and 9.9 ± 0.2 sec (Efnb2 CKO). There was no significant difference between these two groups (t-test, p = 0.18) (Figure 3D). Acute thermal threshold was also examined using a hot-plate. The means latency to withdrawal of a hindpaw at 50°C was 32.2 ± 3.6 sec (Efnb2^fl/fl^) and 31.6 ± 1.7 sec (Efnb2 CKO), at 55°C was 12.8 ± 0.7 sec (Efnb2^fl/fl^) and 14.8 ± 0.5 sec (Efnb2 CKO), respectively. There was no significant difference between these two groups either at 50°C (t-test, p = 0.89) or at 55°C (t-test, p = 0.06) (Figure 3E). Acute thermal threshold was also examined using a cold-plate. The mean time to a paw lifting and/or jumping at 0°C was 79.1 ± 7.8 times (Efnb2^fl/fl^) in 10 minutes and 78.0 ± 5.7 times (Efnb2 CKO). There was no significant difference between these two groups (t-test, p = 0.91) (Figure 3F).

### Ephrin-B2 CKO mutant mice show deficits in inflammatory pain

**Figure 4:**
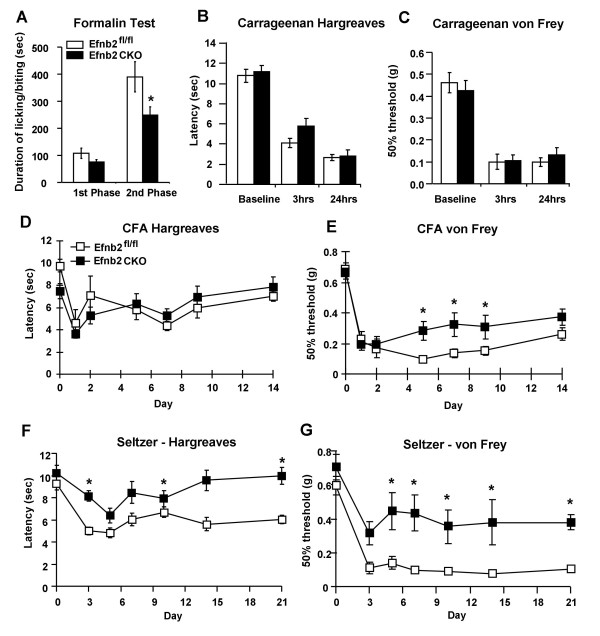
**Both inflammatory and neuropathic pain behavior are attenuated in ephrin-B2 null mutant mice**. **(A) **Two phases (0 - 10 min, 10 - 60 min) of licking and biting behavior after intraplantar injection of formalin were analyzed, the second phase of which is attenuated in Efnb2 CKO mutant mice. **(B) **Intraplantar injection of carrageenan caused identical thermal hyperalgesia in Efnb2^fl/fl ^controls and Efnb2 CKO mice. **(C) **Intraplantar injection of carrageenan caused a long-term mechanical allodynia in both Efnb2 CKO mutants and controls. **(D) **Intraplantar injection of CFA caused identical thermal hyperalgesia in Efnb2^fl/fl ^controls and Efnb2 CKO mutant mice. **(E) **However, intraplantar injection of CFA caused a long-term mechanical allodynia that is attenuated after 4 hours in Efnb2 CKO mutants (two-way ANOVA, * p < 0.05). **(F) **Both Efnb2 CKO mutant mice and Efnb2^fl/fl ^controls showed thermal hyperalgesia after sciatic nerve ligation (Seltzer model). However, thermal hyperalgesia was attenuated in Efnb2 CKO mutants (two-way ANOVA, * p < 0.05). **(G) **Sciatic nerve ligation following the Seltzer procedure caused a long-lasting mechanical allodynia that was attenuated after 5 days of surgery in Efnb2 CKO mutant mice (two-way ANOVA, * p < 0.05).

Tonic responses to intraplantar formalin were observed in formalin test [21]. The first phase (0-10 min) showed no significant difference in pain behavior between the two groups, but the second phase (10 - 60 min) was reduced in Efnb2 CKO mutant mice (t-test, p = 0.048). The mean numbers of lifting and biting of the injected paw was 390.3 ± 56.3 sec (Efnb2^fl/fl^, n = 8) and 248.6 ± 31.4 sec (Efnb2 CKO, n = 11) (Figure 4A). Interplantar injection with the inflammatory agent carrageenan leads to a pronounced thermal hyperalgesia and mechanical allodynia that were maintained over a period of 24 hours. However there were no significant difference showing between the ephrin-B2 CKO mice and floxed littermate controls (Figure 4B and 4C). We also tested inflammatory pain with CFA model. Thermal and mechanical responses were recorded at Day 1, 2, 5, 7, 9 and 14 after the CFA injection. Ten Efnb2 CKO mutant mice and ten Efnb2^fl/fl ^controls were observed with Hargreaves and von Frey. Both groups had developed thermal hyperalgesia and mechanical allodynia 24 hours after CFA injection (Figure 4D and 4E). Both Efnb2 CKO mice and Efnb2^fl/fl ^controls show the similar thermal hyperalgesia response (Figure 4D). However, the Efnb2 CKO mutants displayed overall less mechanical allodynia and significant differences were recorded from 5 days after injection (two-way ANOVA, p < 0.05) (Figure 4E).

### Ephrin-B2 CKO mutant mice show deficits in neuropathic pain

The Seltzer model of partial sciatic nerve ligation was used to assess the alteration in thermal and mechanical sensitivity characteristic of neuropathic pain. Following a Seltzer ligature, both thermal hyperalgesia and mechanical allodynia were developed in both sets of animals (Figure 4F and 4G). However, deletion of ephrin-B2 from nociceptors substantially reduced the mechanical allodynia. Efnb2^fl/fl ^mice showed a substantial decrease in response threshold, to ~ 15% of baseline 3 days after the surgery. In contrast, Efnb2 CKO mutant mice maintained a threshold of ~ 70% of baseline (two-way ANOVA, p < 0.001) (Figure 4G). Also, deletion of ephrin-B2 from nociceptors reduced thermal hyperalgesia. (two-way ANOVA, p < 0.01) (Figure 4F).

### c-Fos expression in spinal cord of ephrin-B2 CKO mutants is diminished in inflammatory pain

**Figure 5:**
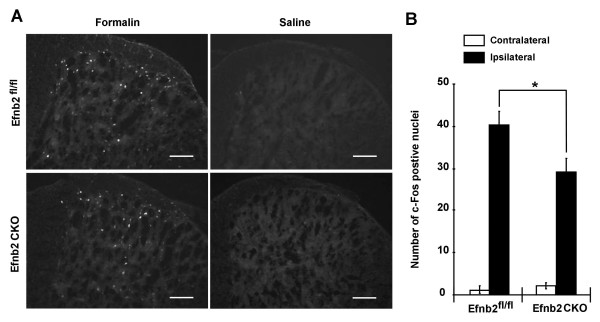
**c-Fos expression was attenuated in the spinal cord in ephrin-B2 conditional mutant mice after formalin injection in the hindpaw**. **(A) **Representative immunostaining for c-Fos in superficial laminae I, II after formalin injection. The right hemicord is showing the cord ipsilateral to the injection of formalin or saline. **(B) **Cell counting for c-Fos positive nuclei in the dorsal horn. Compared to Efnb2^fl/fl ^control mice, the number of positive c-Fos nuclei in the ipsilateral side in Efnb2 CKO mutants is significantly reduced after formalin injection. Scale bar = 100 μm. All data presented as means ± SEM; using students t-test, * p < 0.05.

Proto-oncogene c-Fos is rapidly and transiently expressed in the neurons of spinal cord in response to peripheral noxious stimuli [22,23]. Activation of c-Fos has been used as a neural marker of pain pathways and pain intensity [24]. We examined the expression of c-Fos in the spinal cord after formalin stimulation in the hindpaw (Figure 5A). The result shows that there were 40.3 ± 3.0 (n = 3) c-Fos positive cell nuclei per semi-section in the laminae I and II of spinal cord from Efnb2^fl/fl ^controls and 29.3 ± 3.1 (n = 3) c-Fos positive cell nuclei per semi-section in the spinal cord from Efnb2 CKO mutants, a significant decrease of the formalin induced c-Fos expression in the spinal cord from Efnb2 CKO mutant mice compared to the Efnb2^fl/fl ^controls (t-test, p = 0.046) (Figure 5B).

### NR2B phosphorylation in spinal cord of ephrin-B2 CKO mutants is lost in some inflammatory pain models

Immunoprecipitation (IP) and Western blot of NR2B was performed to establish whether formalin treatment induces tyrosine phosphorylation of NMDA receptor subunit NR2B, and if the extent of the phosphorylation is different in Efnb2 CKO mutant mice and Efnb2^fl/fl ^controls. Thirty minutes after formalin injection in the hindpaw proteins were extracted from the dorsal horn in spinal cords (L3-L5) and immunoprecipitated with NR2B antibody. The result shows that the relative level of phosphorylated NR2B in dorsal horn from the Efnb2^fl/fl ^control mice was remarkably increased (184.2 ± 17.1%) after formalin injection. However, there was no significant increase of NR2B phosphorylation in dorsal horn from the Efnb2 CKO mutant mice (93.1 ± 20.7%) (Additional file 1, S4A). NR2B receptor phosphorylation was also detected after CFA injection in the hindpaw (Additional file 1, S4B). The relative level of phosphorylated NR2B was significantly increased in Efnb2^fl/fl ^controls (138.4 ± 13.8%) 24 hours after CFA injection, but not in Efnb2 CKO mice (108.3 ± 13.0%) (Additional file 1, S4B).

### Microglial activation in spinal cord is attenuated in ephrin-B2 CKO mutant mice, but GFAP and ATF3 immunoreactivity are normal in DRG

**Figure 6:**
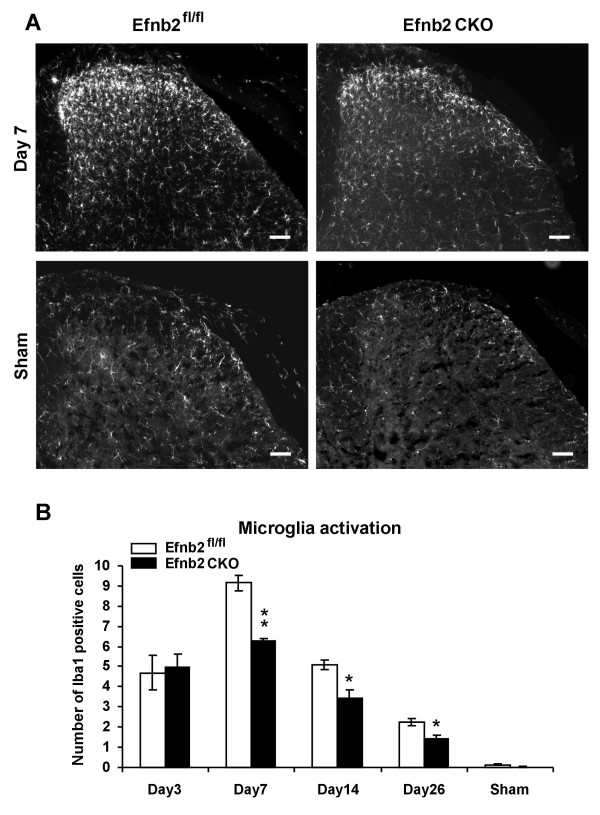
**Increase of immunoreactivity for Iba1 was attenuated in the spinal cord in ephrin-B2 conditional mutant mice in a Seltzer neuropathic pain model**. **(A) **Representative immunostaining for Iba1 in superficial laminae I, II after sciatic nerve partial ligation (Seltzer model). The right hemicord is showing the cord ipsilateral to the sciatic nerve partial ligation. **(B) **Cell counting for Iba1 positive cells in the dorsal horn. The microglia were activated after sciatic nerve partial ligation in both Efnb2^fl/fl ^controls and Efnb2 CKO mice. However, the increase of immunoreactivity for Iba1 was attenuated in Efnb2 CKO mice. Data shown as mean ± SEM, Scale bar = 100 μm. Using students t-test, * p < 0.05; ** p < 0.01.

To investigate the state of glial cell activation in the spinal cord after nerve injury, microglial cells were identified by immunoreactivity for ionized calcium binding adaptor molecule 1 (Iba1) and quantified throughout the lumbar spinal cord. There were similar number of positvie microglial cells in sham animals (0.13 ± 0.04, Efnb2^fl/fl ^controls; 0.02 ± 0.02, Efnb2 CKO) in 10^4 ^μm^2^of the spinal cord section (Figure 6A and 6B). Increasing microglia activation was observed in the ipsilateral dorsal horn of the spinal cord from day 3 onwards following the nerve injury. The number of activated microglia cells per 10^4 ^μm^2 ^(see method) was 4.7 ± 0.8 (Day 3), 9.1 ± 0.3 (Day 7), 5.1 ± 0.2 (Day 14), 2.2 ± 0.1 (Day 26) in Efnb2^fl/fl ^controls, and 4.9 ± 0.6 (Day 3), 6.2 ± 0.1 (Day 7), 3.4 ± 0.4 (Day 14), 1.4 ± 0.2 (Day 26) in Efnb2 CKO mice respectively. Compared to Efnb2^fl/fl ^controls microglial activation in the dorsal horn was significantly reduced seven days after nerve ligation in Efnb2 CKO mutant mice. In the contralateral side, although there was up-regulation of the microglia activation from day 3 onwards, the level is far lower than in the ipsilateral dorsal horn (data not shown). We also examined levels of immunoreactivity for glial fibrillary acidic protein (GFAP) in the spinal cord. After partial ligation of sciatic nerve, the expression of GFAP positive cells in the ipsilateral side of spinal cord significantly increased in Efnb2^fl/fl ^controls and Efnb2 CKO mutant mice compared to sham controls (Additional file 1, S6). However, the increased immunoreactivity of GFAP positive cells in spinal cord was similar between Efnb2 CKO mice and Efnb2^fl/fl ^controls. Expression of Activating transcription factor 3 (ATF3) in the L5 DRG was also investigated 7 days after partial ligation of sciatic nerve. The number of ATF3 positive nuclei obviously increased in contralateral L5 DRGs in both Efnb2^fl/fl ^controls (38.1 ± 5.5%) and Efnb2 CKO mice (34.7 ± 8.3%) (Additional file 1, S5). However, there is no significant difference between the two groups (t-test, p = 0.75).

## Discussion

The present studies demonstrate that presynaptic ephrin-B2 expressed by Nav1.8+ nociceptors has an important role in regulating to the central nervous system in conditions of inflammatory and neuropathic pain. Previous studies, consistent with these data, could not define the cell types involved, or distinguish between pre- or postsynaptic activation of EphB receptors [12,13]. The exclusive pre-synaptic localization of the ephrin-B2 deletion avoids this ambiguity. These results also underline the importance of avoiding floxed exon 1 as a target for Cre recombinase in the generation of conditional knockout mice. Regulatory regions that are associated with transcription factors and the complex machinery involved in controlling gene expression may cause loxP sites to be masked and contribute to the low frequency of DNA excision that we found with floxed exon 1 ephrin-B2 mice. Using identical Cre mice with floxed exon 2 ephrin-B2 mice, gene excision was highly efficient, and allowed us to analyse the role of ephrin-B2 in damage to the central nervous system.

Earlier studies have shown that neutralizing Eph receptor bodies could partially reverse inflammatory pain [9]. Although it is technically difficult to use such approaches to examine long term pain conditions such as neuropathic pain, Eph receptor bodies could prevent or reverse thermal and mechanical hyperalgesia in a chronic constriction injury model of neuropathic pain [12]. These studies implicated EphB/ephrin-B interactions in the onset and maintenance of both inflammatory and neuropathic pain. Kobayashi et al, who showed ephrin-B2 expression up-regulation in DRG and spinal cord neurons in a model of neuropathic pain (spinal nerve ligation), prevented the development of mechanical allodynia with the intrathecal infusion of ephrin-B2 silencing RNAs [10]. This method does not allow us to distinguish between pre- and post-synaptic effects Using conditional knockouts in Nav1.8 nociceptors avoids these difficulties of interpretation.

Ephrin-B2 is involved in intracellular signaling in a range of non-neuronal and neuronal tissues [3,25-27]. Signaling involves small GTPases of the Rho and Ras family the Jak/Stat pathway and the PI3K pathway, whilst reverse signaling involving tyrosine phosphorylation of the ephrin-B2 ligand may also occur [28]. Alterations in the actin cytoskeleton have been associated with changes in synaptic plasticity [29,30]. In addition, within the nervous system, regulation of NMDA receptors through ephrin-B2 induced phosphorylation has been described [31]. Slack et al. also demonstrated that intrathecal infusion of ephrin-B2 in adult rats induces NR2B phophorylation, probably via src family kinases, and EphB1 receptor body infusion prevents NR2B phophorylation in the carrageenan model of inflammation [32].

The deficits in pain induced by tissue damage (formalin model) are associated with a diminished NMDA receptor NR2B tyrosine phosphorylation and a lower level of c-Fos expression in the dorsal horn of the spinal cord. These suggest that, following tissue injury, activation of post-synaptic EphB receptors by pre-synaptic ephrin-B2 is necessary to induce increased in Ca^++ ^currents via NMDA receptors, regulated by NR2B phosphorylation, and consequent spontaneous pain behavior.

The mechanism underlying the enhanced signaling mediated by ephrin-B2 in inflammation and tissue injury is likely to reflect increased ephrin-B2 protein expression in sensitized pain states. In other cell types, for example bone, the induction of ephrin-B2 expression through the activation of transcription factor NFAT has been documented [33]. Many inflammatory mediators - for example bradykinin - are also known to induce NFAT expression, so it is likely that enhanced expression of ephrin-B2 results in NMDA receptor NR2B phosphorylation that potentiates synaptic efficacy and increase calcium flux. Increased noxious input into the dorsal horn reflected by enhanced c-Fos expression in inflammatory pain states is diminished in the ephrin-B2 conditional knockout mice.

There is also evidence that in neuropathic pain caused by nerve crush, ephrin-B2 expression is enhanced with similar consequences for synaptic signaling [10]. In many neuropathic pain models there is also an inflammatory pain component. We found that in the Chung model of neuropathic pain, the cell types involved were not principally Nav1.8-positive neurons, whilst in the Seltzer model, these cells do contribute to some extent to neuropathic pain. In the present studies the Seltzer neuropathic pain models showed deficits in the absence of ephrin-B2, although this was not reflected in ATF3 expressing damaged cells, providing further support to the hypothesis that ephrin-B2 present in DRG cells signals to postsynaptic neurons to induce neuropathic pain. Interestingly, the recruitment of activated microglia, considered an important element in the induction of neuropathic pain was lowered at seven days post-injury, suggesting that signals from ephrin-B2 expressing nociceptors mediated by dorsal horn neurons result in some form of recruitment of activated microglia within the spinal cord. However, the sensitized pain state associated with nerve damage arises considerably before this, suggesting that ephrin-B2 signaling is not the principal mechanism for the initial recruitment of activated microglia.

## Conclusions

In summary, the present conditional ephrin-B2 gene deletion studies confirm a significant role for ephrin-B2 signaling in regulating pain thresholds in inflammatory and neuropathic pain. The mechanisms involved include NMDA receptor phosphorylation as first described in cultured cortical and hippocampal neurons [31] with subsequent enhanced nociceptive input into the dorsal horn. However, in the hippocampus (or elsewhere) a role for presynatic ephrin-B2 has still not been conclusively demonstrated [27], therefore this is the first study to provide evidence in favor of this role. Given the broad range of physiological systems in which ephrin plays an important regulatory role, it is unlikely that targeting ephrin mediated in peripheral pain pathways will provide a side-effect free approach to treating pain. Nonetheless this sensory system provides an excellent model to relate molecular changes in synaptic efficacy to behavioral effects.

## Methods

### Generation of Nav1.8-specific ephrin-B2 null mutant mice

To generate nociceptor-specific ephrin-B2 null mutants, two floxed ephrin-B2 lines; containing either floxed exon 1 [16] or floxed exon 2 [17], were crossed with the Nav1.8-Cre mice [18] to effect ephrin-B2 gene ablation in a defined subset of sensory neurons. The study population contained the homozygous floxed ephrin-B2 gene and one copy of the Nav1.8-Cre allele, whilst homozygous floxed ephrin-B2 littermates were used as controls. Mice were housed with a 12-hr light: 12-hr (lights on at 07:00) dark cycle and maintained under standard condition (21 ± 1°C, food and water ad libitum).

### Genotyping with PCR

DNA was isolated from ear or DRGs. The ephrin-B2 exon 1 wild-type fragment (627 bp), floxed exon 1 fragment (700 bp) and exon 1 mutant fragment (328 bp) were detected by PCR with primer set: a) 5'-AGGGACGCGCAGGGTGAG-3'; b) 5'-CAATGTGTGTCTGTAGCCCCGTTA-3' (Additional file 1, S1). The ephrin-B2 exon 2 wild-type fragment (262 bp), floxed exon 2 fragment (~380 bp) and exon 2 mutant fragment (~320 bp) were detected by PCR with primer: a) 5'-CTTCAGCAATATACACAGGATG-3'; b) 5'-TGCTTGATTGAAACGAAGCCCG-3'; c) 5'-AATACTGTTACTACAGGGTCC-3' (Figure 1A). The Nav1.8-Cre fragment (461 bp) was detected by PCR with primer: d) 5'-TGTAGATGGACTGCAGAGGATGGA-3'; e) 5'-AAATGTTGCTGGATAGTTTTTACTGCC-3' (Figure 1A).

### Genotyping with southern blot

A 3' external probe (709 bp), which was amplified by PCR from C57BL/6 mouse genomic DNA with a forward primer (5'-GCTTGAGTTGAAACGCGGAGG-3') and a reverse primer (5'-CACCTCGCTGCTGATGGAGA-3'), was used to detect the Nav1.8 allele (Additional file 1, S2). A 5' arm external probe (790 bp), which was amplified by PCR from C57BL/6 mouse genomic DNA with a forward primer (5'-ATGTAGGTTTGCACTGGCGAAT-3') and a reverse primer (5'-GAGGTATTGTGGTGGGCAAATC-3'), was used to detect the ephrin-B2 allele (Additional file 1, S3). The probes were labeled with [α-^32^P]dATP (Amersham) using Prime-It II Random Primer Labeling Kit (stratagen) and purified with QIAquick Nucleotide Removal Kit (Qiagen). Southern blot analyses were carried out by standard protocol [34]. Briefly, DNA was extracted from tail or DRG. 10 μg restricted DNA was separated on a 0.7% agarose gel, and transferred onto Hybond-N nylon membrane (Amersham). Filters were hybridized with ^32^P-radiolabeled 5' probe or the 3' probe, then were washed at decreasing concentrations of SSC with the final wash being in 0.1× SSC/0.1% SDS at 65°C and exposed to X-R Kodak film for 48 hours. Southern blot hybridization of *Bam*HI-digested genomic DNA with the 3' probe yielded a 6.7 kb wild-type band and 7.2 kb targeted band (Additional file 1, S2). Southern blot hybridization of *Hind*III-digested genomic DNA with the 5' probe yielded a 6.3 kb wild-type band, 4.1 kb floxed band and 6.1 kb knockout band. Southern blot hybridization of *Hind*III/*Eco*RI-digested genomic DNA with the 5' probe yielded a 4.2 kb wild-type band, 4.1 kb floxed band and 4.8 kb knockout band (Additional file 1, S3).

### Immunohistochemical analysis

Mice were deeply anesthetised with pentobarbital (140 mg/kg) and transcardially perfused with heparinised saline (0.9% NaCl) followed by 4% paraformaldehyde (PFA) in 0.1 M phosphate buffer (PB), pH7.4. The lumbar spinal cords and DRGs were dissected and post-fixed in 4% PFA overnight at 4°C. The tissue was transferred into 20% sucrose in PB for 24 hours at 4°C, and then blocked in OCT compound (BDH Essex, UK) in liquid nitrogen. Transverse sections were cut at 11 μm (DRGs) and 15 μm (lumbar cords) thickness on a Cryostat, and then mounted onto Superfrost plus slides. The slides were washed three times for 5 min each with 0.01 M phosphate buffer solution (PBS). After blocking in 10% goat serum in PBS for 1 hour, the sections were incubated with the primary antibodies overnight at room temperature. After three times washes with PBS, bound primary antibodies were detected by incubating with the secondary antibodies at room temperature for 2 hours. The slides were then washed with PBS for three times and mounted using Fluorsave. The following primary antibodies were used: rabbit anti CGRP (1:2000, Sigma C8198 ); lectin IB4-biotin (1:500, Sigma L 2140); rabbit anti neurofilament 200 (N200) (1:1000, Sigma, UK, N4142); mouse anti βIII tubulin (1:1000, Abcam Ab7751); rabbit anti c-Fos (1:2500, Calbiochem); rabbit anti Iba1 (1:1000, Wako); rabbit anti GFAP (1:1000, Dako cytomation, Denmark); rabbit anti ATF3(1:500, Santa Cruz sc-188). The following secondary antibodies were used: goat anti rabbit IgG Alexa fluor 488 (1:1000, Molecular Probes A11034); goat anti rabbit IgG Alexa fluor 546 (1:1000, Molecular Probes A11035); extra avadin TRITC (1:500, Sigma E3011); goat anti mouse IgG Alexa fluor 488 (1:1000, Molecular probes A11029); donkey anti goat TRITC (1:200, Stratech 705-025-147). All antibodies were diluted to working concentrations with PBS with 1% (w/v) Triton X-100 and 0.01% (w/v) sodium azide. Five sections from each sample were photographed using a Zeiss Axionplan 2 microscope and a Zeiss Axiocam HR. Photo acquisition used Axiovision 40LE version 4.6 software. All neuronal cells with a clear nuclear profile in DRGs were counted using the ImageJ image processing and analysis program. For analysis of the nociceptive terminal staining (CGRP and IB4) in the spinal cord, the area and intensity of the immunoreactive positive fibres were measured. For analysis of the GFAP staining, quantitative assessment of immunoreactivity was carried out by determining the immunofluorescence intensity within a fixed area of the dorsal horn. Five L4-5 sections from each animal were chosen at random with the treatment blinded. 4 boxes measuring 10^4 ^μm^2 ^were placed onto areas of the lateral, central and medial dorsal horn. Mean grey intensity of this area was determined using KS300 software (Axiovision software).

### Behavioral analysis

All behavioral tests were approved by the United Kingdom Home Office Animals (Scientific Procedures) Act 1986 and performed in a Home Office designated room at 22 ± 2°C. Experiments were performed on animals of at least 8 weeks of age as described [35] in rotarod, Hargreaves' apparatus, hotplate, von Frey, Randall-Selitto, and thermal hyperalgesia model induced by carrageenan. The experimenters were blind to the genetic status of test animals. The Complete Freund's Adjuvant (CFA, Sigma) inflammatory model were performed by injecting 20 μl of CFA into the plantar surface of the left paw. The Formalin test was performed by intraplantar injections of 20 μl of 5% formalin. The mice were observed for 60 minutes and the time spend biting and licking the injected paw were monitored. The result was categorized into two phases, the first phase lasting 0 - 10 minutes and the second phase 10 - 60 min. Cold plate behavior was performed as described [36]. We used the Seltzer model to test neuropathic pain. Baseline responses for mechanical and thermal stimulation were obtained according to the protocols for von Frey and Hargreave's methods. Animals were anaesthetised using Halothane. An incision was made in the skin of the upper left leg and blunt scissors were used to part the muscle layers to access the sciatic nerve. A tight ligation of between 1/2 - 1/3 of the nerve was made using 6 - 0 mersilk suture (Ethicon, UK). The skin was closed using 4 - 0 mersilk sutures (Ethicon, UK). Responses to mechanical stimulation and thermal stimulation were assessed with von Frey filaments and Hargreaves' at 3, 5, 7, 14 and 21 days after surgery.

### c-Fos immunohistochemistry

Two hours after intraplantar injections of 20 μl of 5% formalin in right hind paw, the ephrin-B2 CKO mice and floxed littermates were deeply anaesthetized and perfused and the sections of spinal cord were stained with anti c-Fos antibody as described above. The level of immunoreactive c-Fos expression was counted in the ipsilateral dorsal horn region to the formalin or saline injection in laminae I and II. And the experimenters were blind to the genetic status of test animals.

### IP and western blots

Lumber dorsal horn tissue was used for the detection of NR2B phosphorylation after formalin or CFA injection. As described previously [32], mice were anesthetized with pentobarbital (140 mg/kg) and the spinal cords were rapidly obtained by hydraulic extrusion. The dorsal horns of the lumbar enlargement were dissected out on ice and were then immediately snap-frozen on liquid nitrogen. They were homogenized in ice-cold modified RIPA lysis buffer containing protease and phosphatase inhibitors (150 mM NaCl, 50 mM Tris, pH 8.0, 1 mM EDTA, 1% v/v Nonidet P-40, 0.5% SDS, 1 mM phenylmethanesulfonyl fluoride, 5 mM NaF, 1 mM NaVO_4_, 10 μg/ml each antipain, leupeptin and pepstatin). The samples were left to homogenize for 2 hours under rotating agitation. The extracts were centrifuged and the supernatant retained. 1 mg of total protein was added to 5 μg of goat anti-NR2B (Santa Cruz) antibody and gently shaken overnight at 4°C. Protein-G sepharose beads were added to the samples and gently shaken for 2 hours at 4°C. Beads were then rinsed in lysis buffer and boiled in loading buffer. Proteins were separated by 10% SDS-PAGE and transferred onto PVDF membrane as described [35]. The membranes were blocked in 5% BSA in TBST (150 mM NaCl, 25 mM Tris-HCl pH 7.6, 3 mM KCl, 0.1% Tween 20) and were incubated in mouse anti p-Tyr (1:2000; Santa Cruz) and rabbit anti NR2B (1:1000, Upstate,) at 4°C overnight. Blot was washed in TBST and incubated with goat anti mouse Alexa 680 (1:20000, Invitrogen) and donkey anti rabbit IR 800 (1:20000, LiCor Biosciences UK) at room temperature for 1 hour. All antibodies were diluted in TBST. Protein bands were visualized and analysed using the Li-Cor Odyssey Infrared Imaging System (Li-Cor Biosciences UK, Odyssey 2.0).

### Microglia and astrocyte activation in the spinal cord

Immunohistochemical techniques were used to determine the occurrence of microglia and astrocyte activation in the dorsal horn. At day 3, 7, 14 and 26 after partial ligation of the sciatic nerve (Seltzer model), mice were perfused and the sections of spinal cord were stained with anti Iba1 antibody and anti GFAP antibody as described above. We used quantitative image analysis of immunoreactivity for Iba1, the expression of which is upregulated following activation of microglia, as a measure of cell activity. Five L4-5 sections from each animal were chosen at random with the treatment blinded. 4 boxes measuring 10^4 ^μm^2 ^were placed onto areas of the lateral, central and medial dorsal horn. The mean number of positive cells was determined within this area [37]. For GFAP staining, quantitative assessment of immunoreactivity was carried out by determining the immunofluorescence intensity within a fixed area of the dorsal horn. Five L4-5 sections from each animal were chosen at random with the treatment blinded. 4 boxes measuring 10^4 ^μm^2 ^were placed onto areas of the lateral, central and medial dorsal horn. Mean grey intensity of this area was determined using KS300 software (Axiovision software).

### Expression of ATF3 in DRG

Seven days after partial ligation of the sciatic nerve (Seltzer model), the ephrin-B2 CKO mice and floxed littermates were perfused. DRG sections were then stained with anti ATF3 antibody as described above. All neuronal cells with a clear nuclear profile were counted using the ImageJ image processing and analysis program. Five L5 DRG sections from each animal were chosen at random with the treatment blinded. All cells stained with the general neuronal marker βIII tubulin and anti ATF3 antibody were counted. The percentage of ATF3 positive neurons in the DRG was then calculated [38,39].

### Statistical Analysis

All data are presented as mean ± SEM. Data were assessed for normality, and normally distributed data sets were compared with two-way analysis of variance (ANOVA). Non-normal data were assessed with Student's unpaired t-test. p < 0.05 was regarded as significant. All calculations were done using SigmaStat 3.5.

## List of abbreviations

DRG: dorsal root ganglion; CFA: complete Freund's adjuvant; NMDA: N-methyl-D-aspartic acid; GFAP, glial fibrillary acidic protein; Iba1: ionized calcium binding adaptor molecule 1; ATF3: activating transcription factor 3; Efnb2: ephrin-B2; CKO: conditional knockout.

## Competing interests

The authors declare that they have no competing interests.

## Authors' contributions

JZ and GY performed experiments and wrote the manuscript. CMC preformed part of behavioral studies. MAN, MCL and KK provided the transgenic mice. IG and JNW designed the experiments and wrote the manuscript. All authors have read and approved the final manuscript.

## Supplementary Material

Additional file 1 **Supplemental material**. Click here for file
