# Supplementary material for: Nociceptor-expressed ephrin-B2 regulates inflammatory and neuropathic pain
Source: Mol Pain. 2010 Nov 8;6:77. doi: 10.1186/1744-8069-6-77 (PMC2992507; doi:10.1186/1744-8069-6-77)
Supplement: Additional file 1 — Supplemental material. [file 1744-8069-6-77-S1.PDF]

Additional Files

Additional File S1

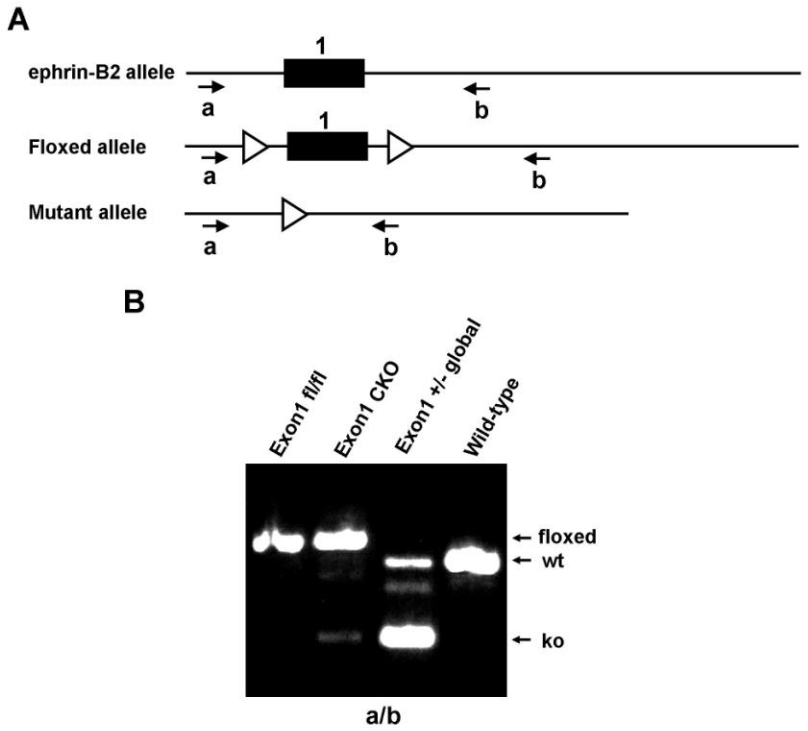

Additional file S1. Genotyping analysis of conditional ephrin-B2 knockout (exon 1 floxed) mice. (A) Diagram showing the ephrin-B2 wild-type locus, the floxed exon 1 locus before and after Cre excision. Exons are represented as numbered boxes. The loxP sites (blank triangles) and the ephrin-B2 PCR primers (black triangles) are indicated. **(B)** PCR analysis for conditional ephrin-B2 knockout (exon 1 floxed) mice. The DRG genomic DNA from homozygous floxed ephrin-B2 exon 1 mice (Exon1 fl/fl), ephrinB2 exon 1 conditional knockout mice (Exon1 CKO), heterozygous ephrinB2 exon 1 global null mice (Exon1 +/- global) and C57BL/6 wild-tpye mice (WT) were examined with PCR. The floxed band (floxed), wild-type band (wt) and knockout band (ko) were amplified with primer a and b. The knock-out band was clearly showed from DRGs in heterozygous global null mice, but it was very weak in homozygous ephrin-B2 (exon 1 floxed) conditional knockout mice.

Additional File S2

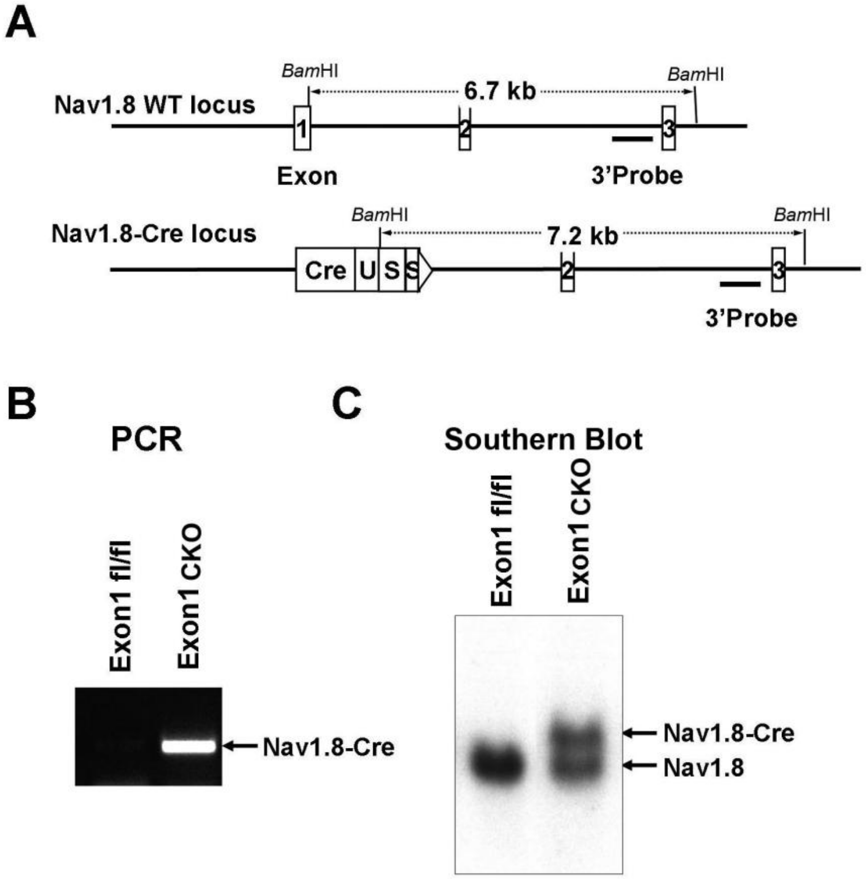

Additional file S2. Genotyping analysis of Nav1.8-Cre in ephrin-B2 exon 1 conditional null mice. (A) Diagram showing the Nav1.8 locus and the Nav1.8-Cre locus. Exons are represented as numbered boxes. Cre, 3'UTR (U), Stop (S), loxP site (blank triangles), 3' probe and *Bam*HI restriction enzyme sites are indicated. **(B)** Analysis of genomic DNA by PCR. PCR was used to detect the genotype for floxed ephrin-B2 exon 1 mice (Exon1 fl/fl) and ephrin-B2 exon 1 conditional knockout mice (Exon1 CKO). The tail DNA was used for PCR (see Materials and methods). The Cre band was detected from ephrin-B2 exon 1 conditional null mutants, but not from floxed controls. **(C)** Analysis of genomic DNA by Southern blot. Genomic DNA was digested with *Bam*HI restriction enzyme and analyzed by Southern hybridization with the 3' probe. This confirms the presence of heterozygous Nav1.8-Cre in ephrin-B2 exon 1 conditional null mice, but not in homozygous Nav1.8 in floxed ephrin-B2 exon 1 mice.

Additional File S3

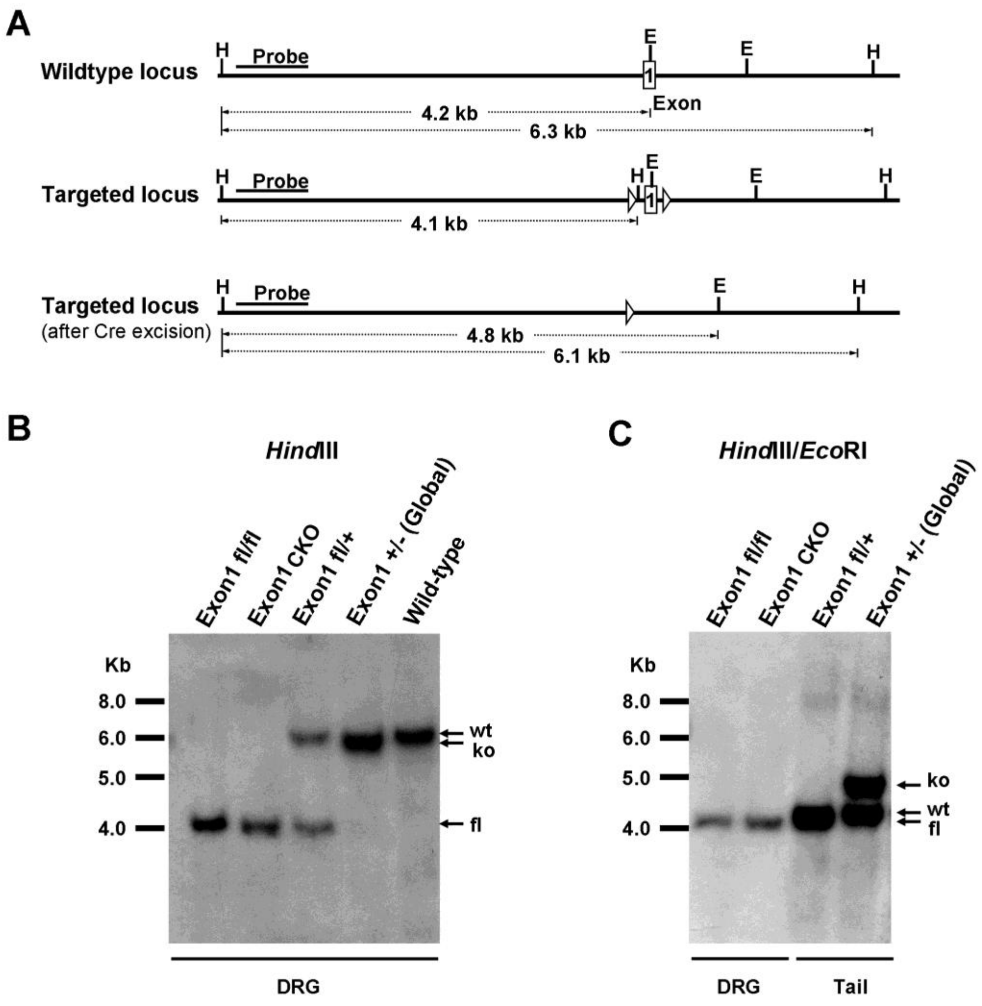

Additional file S3. Southern blot analysis. (A) Diagram showing the ephrin-B2 wild-type locus, the targeted locus before and after Cre excision. Exons are represented as numbered boxes. The loxP site (blank triangles), ephrin-B2 probe, *Hind*III (*H*) and *EcoR*I (*E*) restriction enzyme sites are indicated. **(B)** Digestion of genomic DNA with *Hind*III. The DRG genomic DNA from homozygous floxed ephrin-B2 exon 1 mice (Exon1 fl/fl), heterozygous floxed ephrin-B2 exon 1 mice (Exon1 fl/+), ephrin-B2 exon 1 conditional knockout mice (Exon1 CKO), heterozygous ephrin-B2 exon 1 global null mice (Exon1 +/- global) and C57BL/6 wild-type mice (WT) were digested with *Hind*III restriction enzyme and analyzed by Southern hybridisation with the ephrin-B2 probe. **(C)** Digestion of genomic DNA with *Hind*III and *Eco*RI. The DRG genomic DNA were digested with *Hind*III and *Eco*RI restriction enzymes, and then analyzed by Southern hybridization with the ephrin-B2 probe. The knockout band (4.8 kb) was identified from ephrin-B2 exon 1 global knockout mice. However, it was not found from the conditional ephrin-B2 exon 1 null mutant mice.

Additional File S4

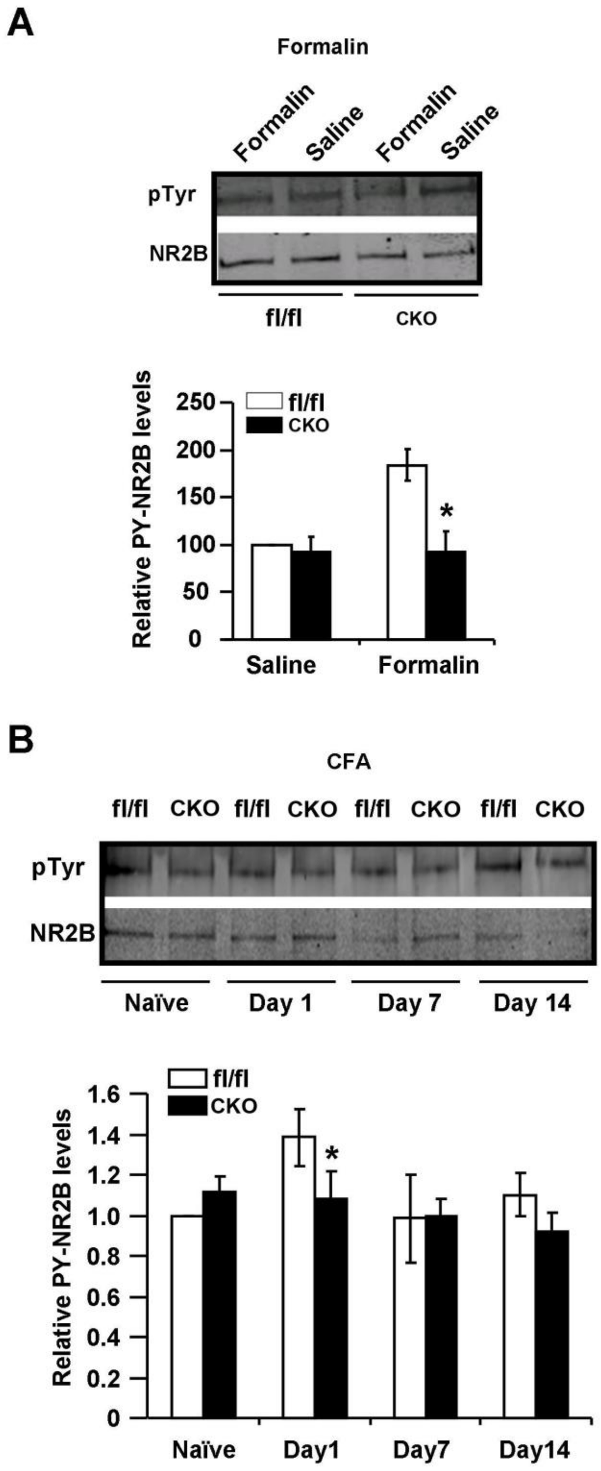

Additional file S4. Formalin and CFA injections in the hindpaw increase NR2B phosphorylation in the spinal cord in Efnb2$^{fl/fl}$ controls (fl/fl), but not in Efnb2 CKO

mutant mice (CKO). (A) NR2B phosphorylation after formalin injection. Thirty minutes after formalin injection in the hindpaw proteins were extracted from spinal cords (L3 - L5) and immunoprecipitated with NR2B antibody. Proteins and antibodies bound to protein A sepharose were separated on 8% SDS-PAGE gels. NR2B phosphorylation was detected with anti phosphotyrosine (pTyr) antibody and the total NR2B was detected with anti NR2B antibody with the same membrane after stripping. The representative blot shows the immuno-reactive bands detected with anti-pTyr antibody (top blot) and anti NR2B antibody (bottom blot). The relative phosphorylation of NR2B protein levels are expressed as a percentage of the vehicle control. **(B)** NR2B phosphorylation after CFA injection. Proteins were extracted from spinal cords (L3-L5) one, seven and forteen days after CFA injection in the hind-paw. The top blot shows the immuno-reactive bands detected with anti-phospho-Tyrosine antibody from the immunoprecipitation with anti-NR2B. The bottom blot shows total NR2B immunoreactivity to compare the amount of proteins in each lane in the same membrane. The relative phosphorylation of NR2B protein levels are expressed as a percentage of the vehicle control. All data presented as means ± SEM; * $p < 0.05$, using student's t-test.

Additional File S5

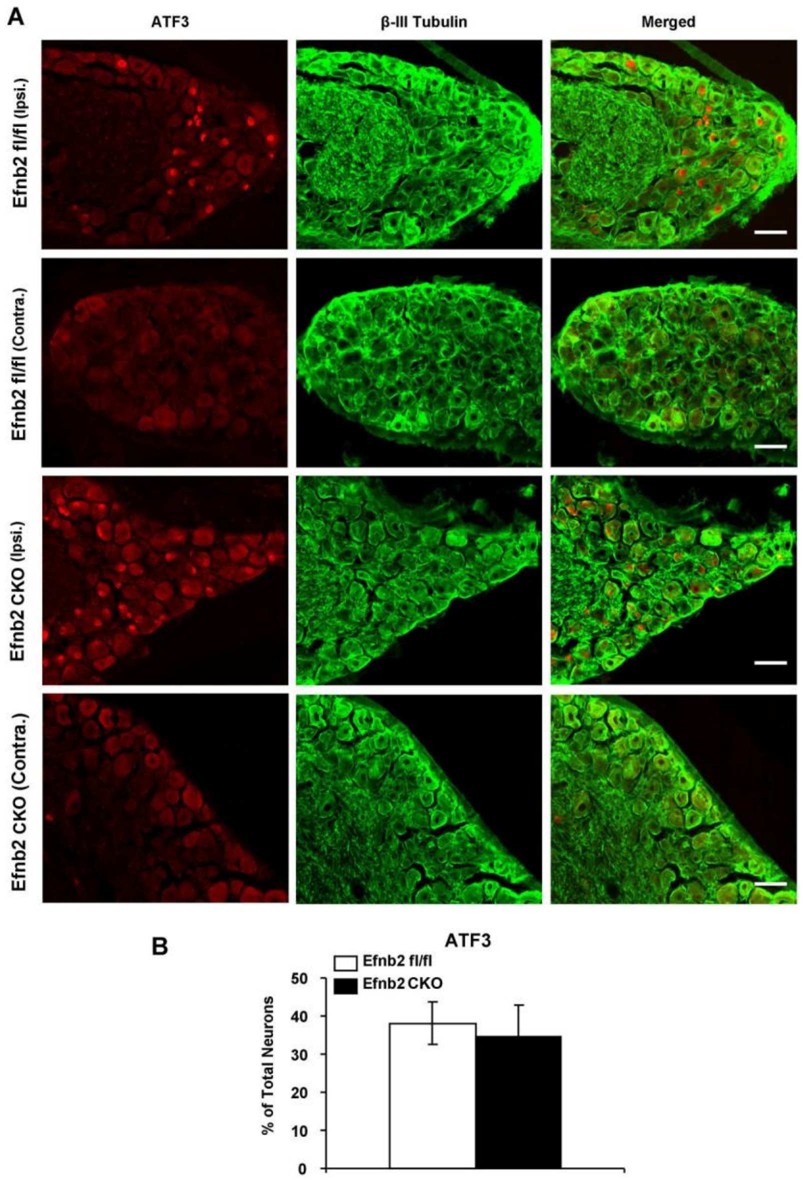

Additional file S5. ATF-3 up-regulation in the DRG in both ephrin B2 conditonal knockout mice (Efnb2 CKO) and floxed littermate controls (Efnb2 fl/fl) after sciatic nerver ligation (Seltzer model). (A) Representative double labeling of ATF-3 (red) and βIII-tubulin (green) in DRG after sciatic nerve partial ligation. **(B)** Quantification of the mean number of positive ATF-3 positive neurons per section in the spinal cord L4 to L5 ipsilateral to nerve ligation. Scale bar = 50 µm. All data presented as means ± SEM.

Additional File S6

Table 1 Glial fibrillary acidic protein (GFAP) immunoreactivity in spinal cord after partial sciatic nerve ligation (Seltzer model)

	Efnb2$^{fl/fl}$ controls		Efnb2 CKO mutants	
	Contralateral	Ipsilateral	Contralateral	Ipsilateral
Sham	--	233.28 ± 18.24	--	218.72 ± 21.32
Day 3	304.63 ± 31.56	368.00 ± 32.78	269.22 ± 22.94	322.72 ± 32.84
Day 7	332.37 ± 5.09	417.07 ± 19.11	297.21 ± 20.03	387.44 ± 32.38
Day 14	293.18 ± 60.00	406.34 ± 86.34	267.72 ± 62.99	372.22 ± 65.62
Day 26	236.31 ± 13.40	335.03 ± 51.64	286.45 ± 10.64	360.11 ± 20.68

Data expressed as mean ± SEM (intensity/10,000 μm^2)
